# Contents, Construction Methods, Data Resources, and Functions Comparative Analysis of Bacteria Databases

**DOI:** 10.7150/ijbs.39289

**Published:** 2020-01-16

**Authors:** Jie Li, Zhuo Chen, Yadong Wang

**Affiliations:** School of Computer Science and Technology, Harbin Institute of Technology, China

**Keywords:** bacterial database, analysis tool, data resource, database construction method

## Abstract

Many bacterial-related databases are developed to meet the researchers' needs of analysis and search for a number of bacterial information. However, these databases have different data resources, construction methods, data formats, and analysis tools. It's difficult for researchers to select appropriate databases and analysis tools to promote their researches. In the paper, we compared the contents, construction methods, data sources, update frequency, scope and scale of data, analysis tools, and features of nine famous bacterial databases: CARD, EffectiveDB, MBGD, MPD, PATRCI, PHI-base, VFDB, gcMeta and SILVA, and help researchers to better make better use of these databases. In addition, we also hope this review can help researchers develop a more comprehensive database and better tools to meet the needs of researchers.

## Introduction

Bacteria not only play an important role in maintaining ecological balance and species diversity 1, but also have a great impact on human activities 2. Although modern medicine has made great progress in the prevention and treatment of bacterial-induced diseases, pathogenic bacteria are still one of the major threats to global public health with the emergence of antibiotic-resistant strains and mutant strains 3, 4. For example, " superbugs " with extensively drug-resistant or totally drug-resistant have been increasing 5.

Bacterial-related database has the potential to provide novel and multiple sources of information for the understanding of infectious diseases, and support for research on disease mechanisms and drugs. For example, researchers can get information such as all the pathogens of a host, all the hosts of a pathogen, evidence of interactions between a pathogen and a host. Researchers also can systematically extract important and specific knowledges from various web-based, free-access bacterial-related databases, and assembled with them into a single database for the study of specific bacterial disease. Databases provide different searching, visualizing, and analyzing tools which can help users find what they want to find, even predict new associations or roles of bacteria. Currently, all kinds of bacterial-related databases were constructed. However, construction methods, data resources, data contents of these databases are different. Moreover, these databases provide different analyzing tools. It's important for users to understand the characteristics, reliability, data size, data content, data source and tool of these databases in order to select the appropriate database for their research. Nine important bacterial databases: CARD 6, EffectiveDB 7, MBGD 8, MPD 9, PATRIC 10, PHI-base 11 , VFDB 12 , gcMeta^13^ and SILVA 14, are introduced in this review. We assessed the content of each database according to the types of data provided by the corresponding bacterial database and the purpose of establishing the bacterial database. We introduced the data scope, scale, and source of each database to determine whether a database is comprehensive or not. We also discussed the search and browse tools provided by different databases and determine whether users can quickly and find the information they want to find in a database. Data analysis is another important service provided by the web-based database. Some databases only provide simple data analysis, others can provide comprehensive data analysis, we compared differences of analysis tools of different databases. The paper is organized as follows. the content, data source, construction method, update frequency, functions, and features of nine important databases are elaborated respectively in Section 2. In Section 3, we compare the nine databases in terms of data sources, construction methods and basic functions, and make a brief evaluation. Finally, we listed the differences of these databases in terms of their content, scope and scale of data, microbiome data, application/tools available, main data sources, update frequency, and data construction methods through a table in the Discussion section, so that researchers can understand the characteristics of different databases more comprehensively.

## Bacterial databases

### The Comprehensive Antibiotic Resistance Database (CARD)

Bacterial resistance refers to the tolerance of bacteria to the action of antibacterial drugs. Once the drug resistance is produced, traditional prevention and treatment methods will be invalidated, thereby increasing the difficulty of treatment and the risk of infection. Due to the abuse of antibiotics, the clinically observed resistance of pathogens continues to increase. Without effective antibiotics, the success rate of major surgery and cancer chemotherapy will be affected. At the same time, due to the failure of traditional treatments, the cost of treatment for patients with drug-resistant bacteria is much higher than for patients infected with non-resistant bacteria. Therefore, bacterial resistance has become a global health crisis 15. Understanding bacterial resistance genes and their circulation between bacteria and patients are especially important for managing the increasingly scarce available antibiotics and guiding the discovery of new drugs.

To meet the research and detection needs of antibiotic resistance genes, CARD is dedicated to collecting genes, proteins and mutation data related to antibiotic resistance 6. Knowledge in CARD is mainly derived from manual text mining. CARD covers a totally of 4236 ontology terms, 2678 reference sequences, and so on. The CARD is updated monthly.

**Figure 1:**
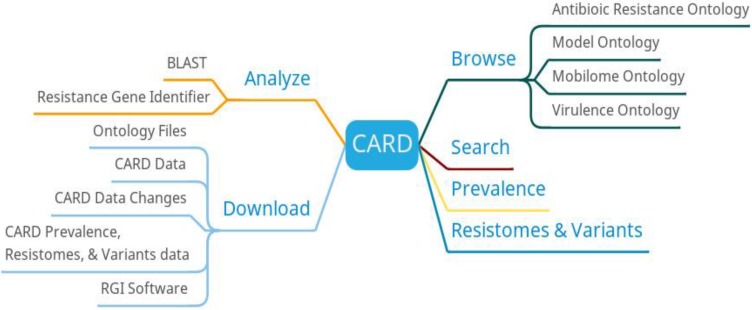
CARD function diagram

In addition to providing browse, search and download, CARD also provides functions such as BLAST search, resistance gene identifier, prevalence and resistomes & variants (Figure 1). The result of BLAST search against the CARD reference sequences is annotated with extra information from CARD. The resistance gene identifier can identify potential resistance genes based on protein, gene, and metagenomic data. The prevalence can get the distribution and proportion of drug resistance genes in 82 important pathogens. The resistomes & variants can be used to detect the resistance group information of the genome, plasmid, and data other of important pathogens.

The high quality of data by manual text mining and model ontology makes CARD to become a more functional resource for antimicrobial resistance data, allowing it to keep pace with the rapidly evolving antimicrobial resistance crisis 16. CARD can detect the missing drug resistance data based on the existing data, actively provide experimental ideas for the researchers and promote drug development.

### The secreted protein alignment analysis database (EffectiveDB)

Proteins synthesized by bacteria in cells need to be transported to certain specific parts or outside for the purpose of survival, reproduction, and spread. Some protein secretion pathways in pathogenic bacteria are mainly used to secrete virulence factors. Therefore, the study of the bacterial secretion system plays a crucial role in understanding the relations between pathogens and virulence and infection-related processes at the molecular level. In addition, it also provides new ways for the diagnosis and treatment of related diseases. Although new molecular experiments and computational methods have driven these areas of development, predictive modeling capabilities for complex host-microbial interactions remain limited and lack of annotation standards associated with secretion 17.

In order to solve this problem, EffectiveDB is developed by collecting relevant data 7. EffectiveDB provides precalculated results for 23 bacterial genomes from the NCBI RefSeq 18 database and 1676 bacterial genomes in the EggNOG 4.0 database, covering 1699 bacterial genome secretion systems and predictions. Updates of the genome repository and all precalculated predictions in the Effective database are automatically conducted quarterly.

**Figure 2:**
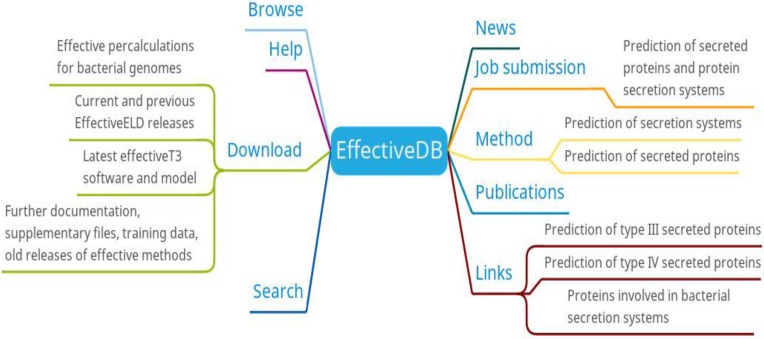
EffectiveDB function diagram

Researchers can browse/search/download the secretion system data and predicted results of bacterial protein secretion based on protein or genome information of different bacteria from EffectiveDB. In addition, researchers also can submit their prediction jobs through job submission or get supplementary information for the predictive models of EffectiveDB through methods (Figure 2). The job submission provides inputting a protein sequence in FASTA format and uploading a FASTA file containing a protein sequence to the prediction of secreted protein and protein secretion systems. You can select EffectiveT3 (predicts Type III secreted proteins based on their signal), T4Spre (predicts Type IV secreted proteins based on amino acid), EffectiveCCBD (predicts Type III secreted proteins based on their secretion), EffectiveELD (predicts secreted proteins based on eukaryotic-like domains) and Predotar (predicts subcellular localization of secreted proteins in the host) prediction model. EffectiveDB also bundles various tools (methods) to predict bacterial secreted proteins and secreted protein systems based on their sequence. Researchers can learn more about the predictive tools (methods) they are interested in through the methods menu.

EffectiveDB is the first bioinformatic resource combing function-based prediction by identification of eukaryotic-like domains and prediction based on signal peptides leading to transport by protein secretion systems 19. The precalculated predictions of secreted proteins and secretion systems by these methods resemble the core of EffectiveDB. In the future, EffectiveDB will integrate more additional bacterial genomes to meet the requirement of researches 7.

### The microbial genome database (MBGD)

With the advancement of gene sequencing technology, the microbial genome database is rapidly expanding. There are now approximately 3,000 complete microbial genome sequences and thousands of incomplete microbial genome sequences in the public sequence database 8. Analyzing these sequence data can help us understand the function, expression mechanism and species evolution of bacterial genes, reveal their pathogenic molecular mechanisms and elucidate the evolutionary relationship between bacteria and the internal structure of the genome.

With the exponential growth of gene data in the past two decades, how to effectively manage, analyze and use existing gene data has become particularly difficult and important. To solve this problem, MBGD (http://mbgd.genome.ad.jp) uses a hierarchical clustering program DomClust to perform a large-scale orthologous comparative analysis of bacteria 20. MBGD first obtained raw gene data from NCBI's GenBank, RefSeq, and DNA Data Bank of Japan (DDBJ) Gene Trek in Prokaryote Space (GTPS) 21. Then, sequence similarity is calculated by the BLAST program, and finally comparative analysis is performed. The functional categories of each orthologous group category are determined according to the rules of majority voting. MBGD includes 6318 genomic data covering 5861 bacteria, 254 archaea, and 203 eukaryotes. MBGD is updated two times per year.

**Figure 3:**
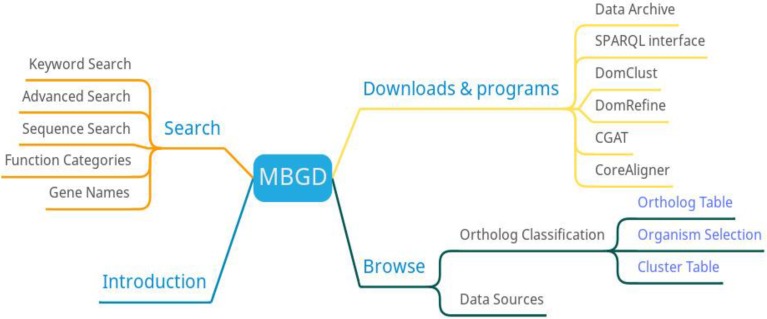
MBGD function diagram

MBGD has three main functions: search, orthologous classification, and download (Figure 3). Researchers can search the information of microbial genomes through function category list, gene name or sequence. MBGD provides a homologous gene table, organism selection, and clustering table to view homologous gene information. In addition to providing data downloads, MBGD also provides comparative genomic software downloads.

MBGD provides orthologous relationships among microbial genomes published so far as a basis for comparative analysis of either closely related or distantly related genomes. One of the features of MBGD is having all-against-all similarities among all the translated sequences of the stored genomes. Besides than researchers can create an ortholog table from any specified set of genomes. In the future, MBGD will develop more effective applications to meet the demand for handling and utilizing large-scale genomic data through comparative analysis 22.

### Mypathogen database (MPD)

In the past, research on microorganisms such as bacteria was mainly based on pure culture. However, this method is not only expensive but also covers only 0.1% to 1% of microorganisms in the environment. In order to solve this problem, metagenomic technology is developed to directly extract total DNA from environmental samples and obtain new functional genes and biologically active substances, thereby freeing the traditional species boundaries and revealing the law of life movement at a higher level. However, the lack of an effective data management system and metadata that can be described not only increases the workload of analyzing data but even makes the data misused.

To solve the problem of data dispersion and inconsistency between resources, MPD was developed by the Bioinformatics Department, State Key Laboratory for Infectious Disease Prevention and Control, Chinese Center for Disease Control and Prevention. MPD aims to provide researchers with the ability to search, download and share bacterial genomic data to facilitate clinical and epidemiological studies at the Chinese Center for Disease Control and Prevention 9. MPD has 72311 data records, in which including 41935 genomes of bacterial strains and 28950 metagenomic data from human and environmental samples. The data in the MPD are mainly from public databases and user uploads. First, MPD obtains genetic data from NCBI 23, Ensemble 24, and EMBL-ENA 25, and metagenomic data from HMP 26, MG-RAST 27, Meta-hit 28, and Imicrobe 29. Then extracts background information of these data for sequence filtering to ensure data non-redundancy. MPD allows users to upload data files in FASTQ, FASTA or TXT formats, and users are free to choose whether or not to public data. The data of MPD is updated yearly.

**Figure 4:**
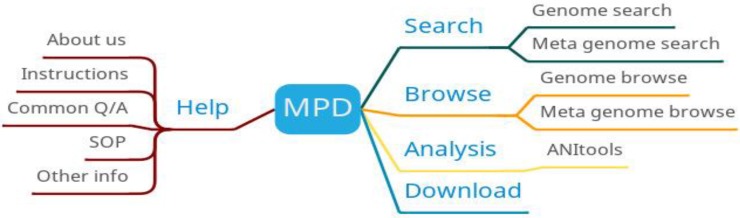
MPD function diagram.

MPD provides four main functions: search, browse, analysis and download (Figure 4). The search function is mainly divided into genome search and metagenomic search, and the search results are displayed in the form of a table. The download function provides downloads of MPD data manipulation tools to help users complete data upload and download. MPD also provides online analysis tools to compare bacterial genomes and calculate their average nucleotide identity (ANI).

As a publicly available bacterial genomic and metagenomic data resource, MPD plays a critical role in filtering manually generated metadata from various resources. As more data are integrated and related services going mature, MPD is expected to develop into a global pathogen genomic and metagenomic data resource 9.

### The Pathogen System Resource Integration Center (PATRIC)

With the growth of biological data, the limitations of traditional research methods in the collection, storage and use of data are becoming more and more obvious. Therefore, the bioinformatics formed by the combination of life science and computer science has emerged. Bioinformatics uses computer technology to collect, process, store and analyze a large number of complex biological data and reveals the mystery of biology.

PATRIC (https://www.patricbrc.org/) is one of the four largest bioinformatics resource centers in the United States dedicated to collecting comprehensive bacterial biodata 10. PATRIC includes bacteria, archaea, virus, and eukaryotic host genomic data. PATRIC regularly obtains antimicrobial resistance, genomes, genomic features, pathways, protein families, specialty genes, and transcriptomics data from different data sources. Then using PATRIC annotation service, GenBank, and RefSeq to annotate these data and storing in the PATRIC database 30. PATRIC is updated and incorporate data into PATRIC on a monthly basis.

**Figure 5:**
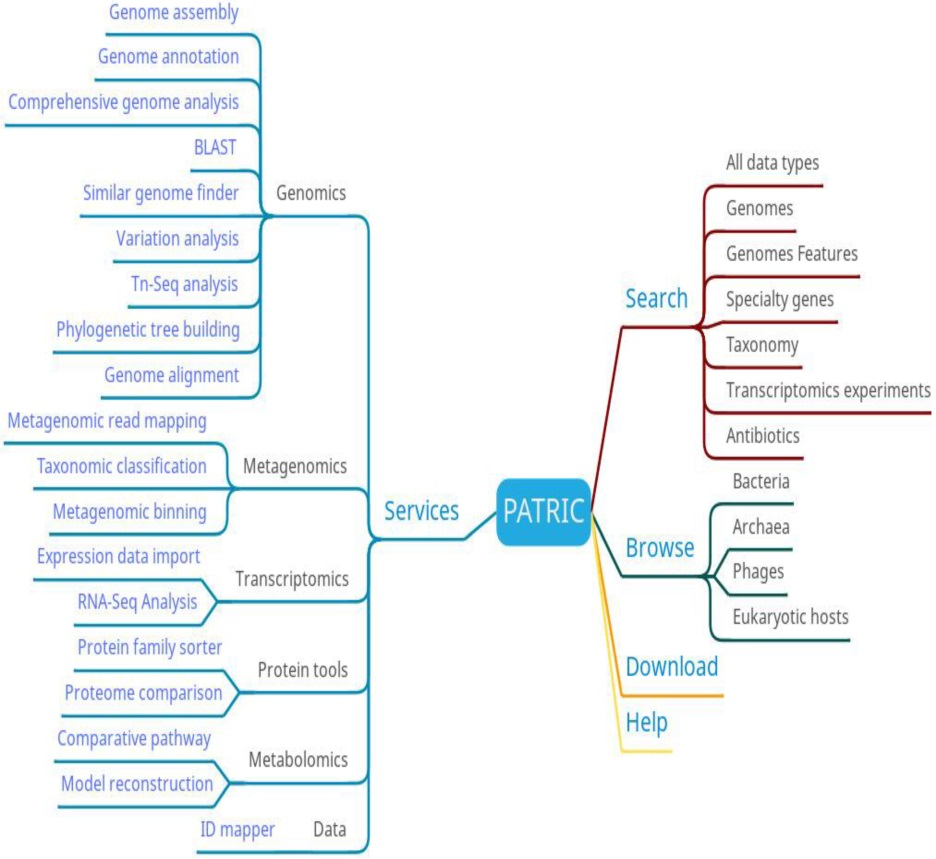
PATRIC function diagram.

The main functions at PATRIC include searching, browsing, analyzing and downloading (Figure 5). Users can browse PATRIC data based on species. They can also search for data by full data type, genome, genomic characteristics, specific genes, species, transcriptomics experiments, and resistance genes. The analytical function is particularly comprehensive, covering genomics, metagenomics, transcriptomics, protein tools, metabolomics, and data online analysis tools, which can meet a variety of bacterial research needs. At PATRIC, researchers can upload their private data and analyze it using high-throughput services, and compare it with other public databases using visual analytics tools.

As a comprehensive bacterial database, PATRIC not only contains comprehensive and complete data, but also covers various online tools. It is one of the rare comprehensive databases, which satisfies the requirements of various experiments. PATRIC will increase efforts aimed at making data and services more useful for clinical researches, modeling, and support in the area of therapeutics.

### The pathogen-host interaction database (PHI-base)

Pathogen-host interactions reflect the infectivity and pathogenicity of pathogens, and research into pathogen-host interactions can reveal pathogen infection, survival, and ways of interfering with host cells, providing ideas for prevention and treatment of diseases. With the advent of molecular cloning technology, functional analysis of genes that determine host-pathogen interactions has become feasible. But the increase in the amount of these data and the excessive dispersion of storage make it difficult for researchers to use them effectively.

To solve this problem, the pathogen-host interaction database (PHI-base, www.phi-base.org) collects experimentally validated pathogenic, virulence and effector gene data from the automated plant pathogens and host species 11. PHI-base has 12467 pathogen-host interaction data. PHI-base's raw data is derived from documents searched from PubMed/MEDLINE and Web of Science, followed by manual text mining into tables. Nucleotide and protein sequences, EC annotations are from EMBL sequence databases and Gene Ontology annotations, respectively. PHI-base also provides external links to PubMed, the digital object identifier for the curated article, NCBI Taxonomy database, and UniProt as the main information sources of data. PHI-base is updated twice a year.

**Figure 6:**
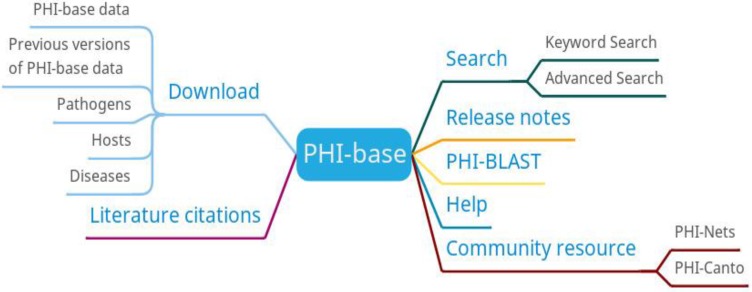
PHI-base function diagram.

The main functions of PHI-base are search, PHI-BLAST, and download (Figure 6). Users can search the information of genes, diseases, hosts, pathogens, anti-infective modes, manifestations, experimental techniques, and host targets. PHI-BLAST allows users to perform BLAST searches based on PHI-base data.

PHI-base's data comes from manual text mining, which guarantees the quality of the data, but it also has the disadvantage of slow update speed. And, compared to similar databases, there is no online tool for predicting pathogen-host interaction relationships at PHI-base.

### The Virulence Factor Database (VFDB)

Bacterial virulence factors, the substances that make up the virulence of bacteria, determine their pathogenic capacity and mechanisms, so an in-depth understanding of virulence factors can provide new ways to treat and prevent diseases caused by bacteria. For this purpose, VFDB was established in 2004 to collect and provide the latest bacterial pathogen virulence factor data 12.

The core data set of VFDB contains 32 bacteria and 575 virulence factors. These core data were first obtained by collecting the virulence factors and bacterial information in PubMed's papers and subsequent review papers, and then using Perl scripts to extract virulence factors and bacterial genome information from GenBank and NCBI databases, respectively. Finally, these data are annotated using RefSeq format labels (including gene ID, reference sequence library, reference sequence library corresponding ID, related genes, products, virulence factors, virulence factor IDs, pathogens, DNA sequences, and protein sequences) 31. VFDB is updated irregularly.

**Figure 7:**
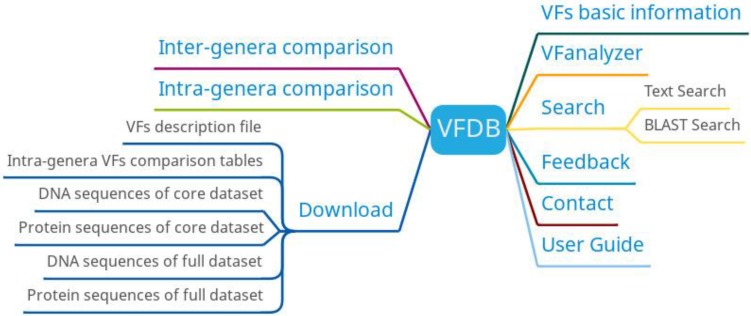
VFDB function diagram.

VFDB has six main functions: basic information of virulence factors, intra-genera comparison, inter-genera comparison, search, VFanalyzer and download (Figure 7). Basic information on bacteria and their associated virulence factors can be found on the virulence factor basic information page. Researchers can get different information of different bacteria with the same virulence factor by intra-genera comparison and predict virulence factors based on bacterial genome information uploaded by users using VFanalyzer.

There are several public bacterial genomic resources providing VF services, but most of them are based on the dataset of VFDB and depend solely on BLAST searches. In addition, VFDB provides VFanalyzer to identification VFs which can reduce the workload of researches. In the future, VFDB will focus on improving VFanalyzer and developing a new bacterial VF prediction method.

### The Global Catalogue of Metagenomics (gcMeta)

With the progress of scientific research, it has been found that simply studying a certain direction cannot explain all biomedical problems. Scientists have put forward from a holistic perspective to study the cellular structure, gene, protein and their interactions among bacterium. At the same time, the next-generation sequencing technology has accelerated the development of meta-omics. But rapid development also brings many challenges. First, the inconsistency of standards between different research projects makes it difficult to compare and analyze these data. The second challenge is the lack of a public data platform capable of comprehensive collection, collation, and open access. The last challenge is the lack of data analysis platforms that can be scaled at GB or even TB levels. To address these challenges, gcMeta(https://gcmeta.wdcm.org) collects data from CMI and global microbial research projects and provides massive data analysis capabilities to meet research needs.

**Figure 8:**
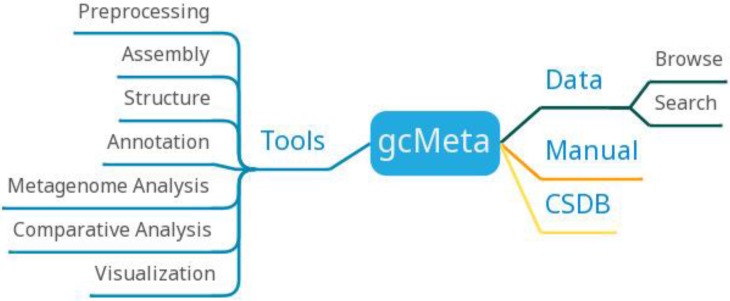
gcMeta function diagram.

gcMeta has archived a total of 126602 samples, 153271 sequences, 146696 experiments, and 77682 reports, hosting more than 120 TB of sequencing data. The data of gcMeta mainly comes from open data sources such as MG-RAST, EBI metagenomics, and HMP and ongoing research projects such as CAS-CMI. The data of gcMeta are mainly divided into 'Study', 'Sample', 'Experiment' and 'Sequence'. 'Study' can be divided into several 'sub-studies' and is related to 'Sample' by the 'Study ID'. 'Sample' is referenced to 'Experiment'. 'Experiment' is further referenced to sequence information. The 'Sequence' includes the sequencing methods and strategies and the processing of the sequencing results. GcMeta is updated monthly.GcMeta provides 90 tools that can be divided into preprocessing, assembly, structure, annotation, metagenome analysis, comparative analysis and visualization (Figure 8). Users can download the corresponding tools to analyze the data according to their own needs. GcMeta updates data once a month.

GcMeta serves as a microbial meta-omics data database, collects data from open data sources and ongoing experimental projects according to international standards, and supports comparative analysis of data from different projects. In the future, gcMeta will further add and integrate data from other open data sources and provide customized analysis workflows to users. In addition, GCM sequencing results will be integrated to provide a more accurate annotation of metagenomic data. In a word, gcMeta will continue to improve to meet the needs of meta-omics research.

### High Quality Ribosomal RNA Databases (SILVA)

Ribosomal RNA (rRNA) is widely used in nucleic acid-based microbial diversity, taxonomy and phylogenetic reconstruction. With the emergence of a new generation of sequencing technology, the amount of rRNA data continues to grow. The storage, retrieval, comparison, and analysis of massive data need to be solved urgently. To solve these problems, SILVA (https://www.arb-silva.de/) collects and checks rRNA data and provides a variety of classification methods and the latest naming methods for both the small subunit rRNA gene (SSU) and the large subunit rRNA gene (LSU). SILVA also provides precise phylogenetic guidance trees in each release.

SILVA has 9470435 SSU Parc, 1312673 LSU Parc, 4945070 SSU Ref, 357845 LSU Ref, 659046 SSU Ref NR 99 and 123524 LSU Ref NR 99. SILVA data mainly come from EMBL-EBI/ENA. SILVA is named according to the release number of EMBL. Each release of SILVA data is divided into two data sets, Parc and Ref. Parc contains all the data in SILVA, Ref contains only high quality and nearly full-length sequence data. SILVA is updated annually according to the update frequency of the EMBL database.

**Figure 9:**
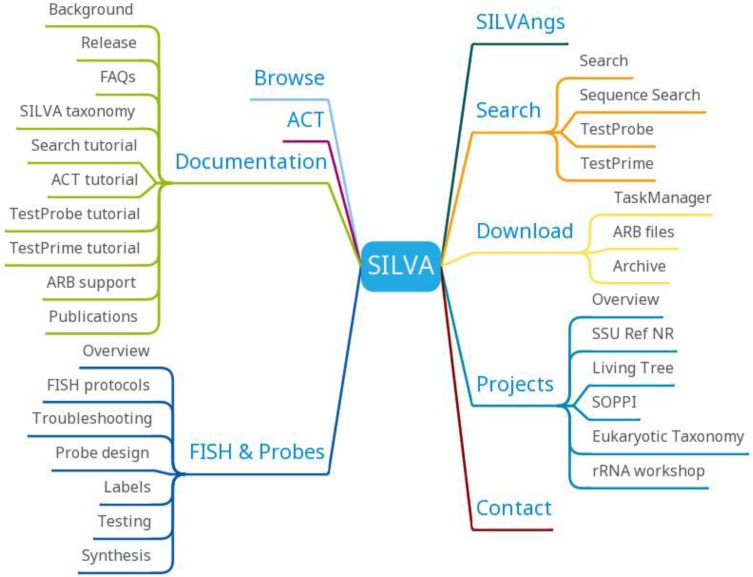
SILVA function diagram.

SILVA provides SILVAngs, SILVA Alignment, Classification and Tree (ACT) Service, SILVA Tree Viewer and ARB tools (Figure 9). SILVAngs is a data analysis service for ribosomal RNA gene (rDNA) amplicon reads from high-throughput sequencing (next-generation sequencing (NGS)) approaches based on an automatic software pipeline. The SILVA ACT service combines alignment, search and classify as well as reconstruction of trees in a single web application. The SILVA Tree Viewer is a web application to browse and query the SILVA guide trees. The software package ARB represents a graphically-oriented, fully-integrated package of cooperating software tools for handling and analysis of sequence information.

As a high-quality ribosomal RNA database, SILVA will provide a more accurate classification of candidate taxonomic units and more rapid and accurate phylogenetic guidance tree generation in the future. SILVA will provide a more stable perspective for the further maintenance and development of the databases and services.

## Database Comparison

### Comparison of data sources

**Figure 10:**
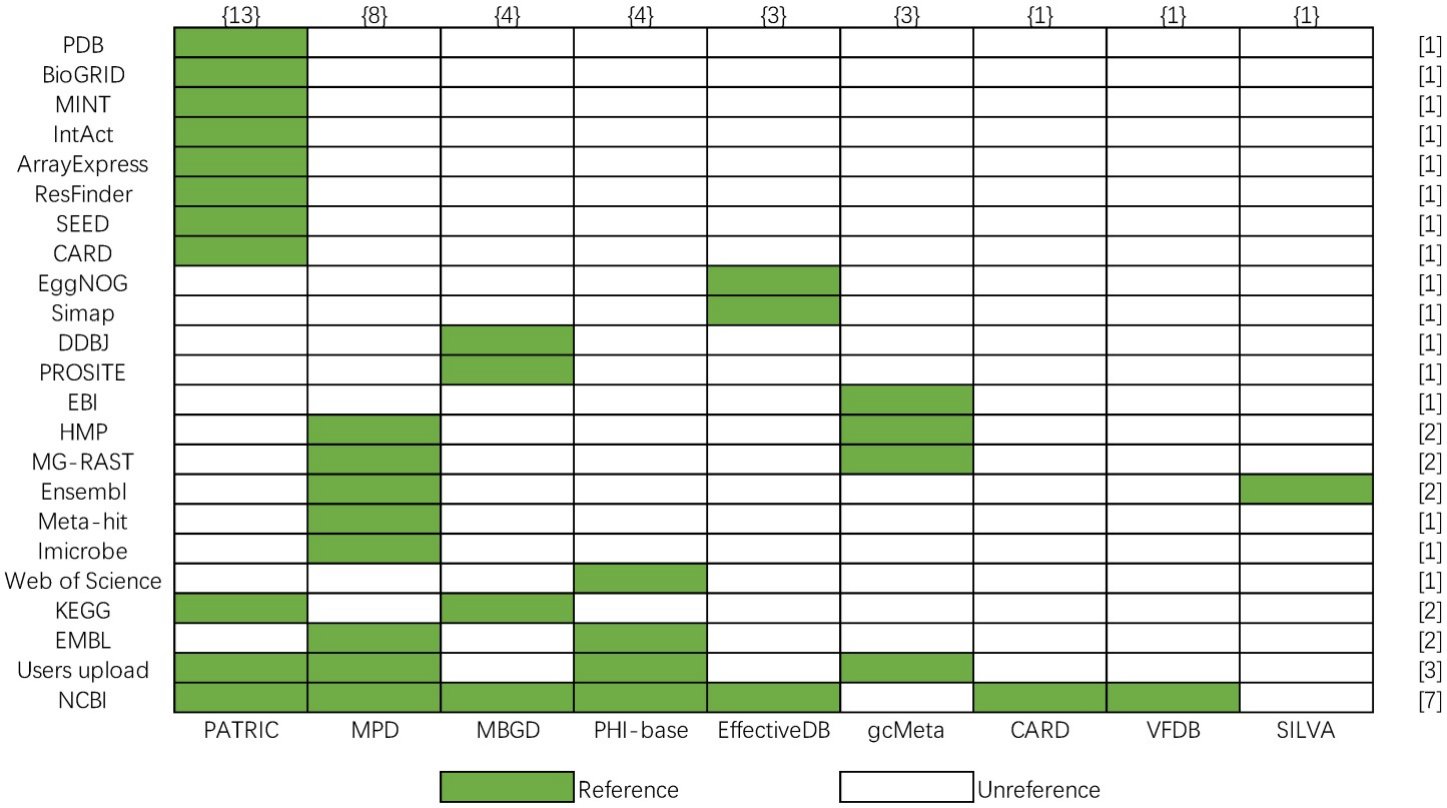
Data source matrix of nine bacteria databases.

Most bacterial databases are based on the known reference databases such as pathway database KEGG 32, protein databases MINT 33, literature resource Web of Science. However, the data sources referenced by the above nine bacterial databases are different. In order to show more clearly their differences in data sources, a matrix of the data source is shown, and the number of data sources is calculated in Figure 10, whose row and column represent the number of reference databases (y-axis) and bacterial databases (x-axis), respectively. The color legend indicates whether data in the database is from a certain reference database. The number in the brace indicates the number of data sources referenced by the corresponding bacterial database, and the number in square brackets indicates the number of times the corresponding data source is referenced. According to Figure 10, PATRIC cites the most data sources in nine bacterial databases. The most frequently cited data source is NCBI, which provides the basic gene sequences, protein sequences, and species data for databases. And PATRIC, MPD, PHI-base, and gcMeta combine user's data with web public data as their data. The comparison shows a good picture of the data redundancy between the various databases.

### Comparison of construction methods

**Figure 11:**
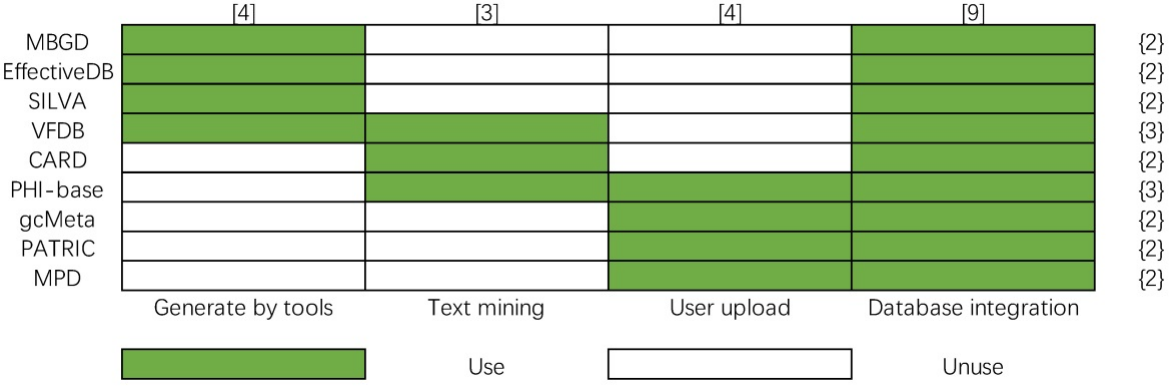
Data construction method matrix of nine bacteria databases.

Four main methods are used to construct bacterial database: text mining, user upload, database integration, and using effective predictive tools to generate data. In order to show more clearly their differences in database construction methods, a matrix of database construction methods is made, and the number of data construction methods is calculated in Figure 11, whose column and row represent the method of database construction (x-axis) and bacteria databases (y-axis), respectively. The color legend indicates whether the database construction method is used by a certain database. The number in the brace indicates the number of data construction methods used by the corresponding bacterial database, and the number in square brackets indicates the number of times the data construction method is used. Database integration is a fast and effective way to construct big bacterial databases, so most bacterial databases are built in this way. However, the problems that need to be solved in building a database by this method are inconsistent data format, eliminating redundant contents, judging inconsistent content, etc. from different databases. The text mining method is used by PHI-base, CARD, and VFDB. The problem of text mining technology is that it is difficult to guarantee the reliability of automatically extracted knowledge. The knowledge-based on text mining needs further manual checking. Uploading data by users is another important way to extend the contents of databases, it is used by PHI-base, gcMeta, PATRIC and MPD. The fourth way to build a database is to take the results of some prediction tools as the contents of the database. It is also difficult to guarantee the reliability of the knowledge generated by prediction tools.

### Comparison of basic functions

The functions of nine databases are compared in terms of browse, search, download, help, and online analysis tools. The comparison results are shown in Table 1. The symbol'√'indicates the function is provided in the corresponding database. The number in the bracket is the number of online analysis tools. From Table 1, it can be seen that the browse, search and download functions appear as the most common functions in all databases. Most databases' browse functions are based on species and search functions are based on keywords or fields. For example, CARD data is stored on ontology, so these functions are also based on ontology. MBGD focuses on ortholog identification, paralog clustering, motif analysis, and gene order comparison, so its functions are also based on these points of view. Most databases provide one or two online analysis tools. MBGD and gcMeta lack the relevant online analysis tool, and yet PATRIC has 19 online analysis tools in genomes, metagenomics, transcriptomics, protein, metabolomics, and so on. CARD lacks the help function to guide users. Although these databases have become mature on their basic functions, there is still a lack of data depth display and analysis tools, such as high-performance genomics data visualization, exploration, analysis tools based on machine learning and statistical methods, and so on.

## Conclusion

In this review, we introduce 9 important bacteria databases in data composition, functions, tools, data sources and so on. These databases are classified by data types: antimicrobial resistance, metagenomics, comparative genomics, pathogen-host interaction, secreted proteins, secreted protein systems, virulence factors, meta-omics, and rRNA. Besides that, we also provide some comparisons of data sources and basic functions that should be helping to develop new databases and improve existing analysis tools. The summary of these databases is shown in Table 2. According to this table, we can see the differences between different databases are reflected in their different focuses and purpose. CARD is designed to collect antimicrobial resistance data and offer some tools for antimicrobial resistance researches. EffectiveDB and MBGD not only integrate data from other open sources but also offer information on secreted proteins and microbial orthologous comparative generated by tools, respectively. Moreover, MPD provides metagenomic data from other databases and users. Gc-Meta collects meta-omics data from open data sources and ongoing projects. PHI-base is a publicly available database that collects pathogen-host interactions using text mining technology. Because of the accuracy and comprehensiveness of VFDB data, many databases use VFDB as basic data set for building their virulence factor data set. SILVA provides high-quality ribosomal RNA data. As a comprehensive bacteria database, PATRIC has been considered an effective platform for bacteria researches. For instance, PATRIC provides seven data types and many services covering genomics, metagenomics, transcriptomics, protein tools, metabolomics, and data.

Altogether, we hope that the review help researchers quickly understand the characteristics of different bacterial databases, select appreciate tools or develop a more comprehensive database and more effective tools to solve challenging problems in the bacterial research area.

The bacterial database is the basis of bacterial researches. Making full use of bacterial data not only provides insight into the mechanisms by which bacteria cause disease but also helps us develop new treatment methods and drugs of bacterial diseases. However, there are still many challenging issues to be addressed, such as efficient data collection, data format uniform, construction of complex data networks, more efficient data analysis tools, a more user-friendly user interface and so on.

Currently, there are abundant bacteria databases that cover antimicrobial resistance, metagenomics, comparative genomics, pathogen-host interaction, secreted proteins, secreted protein systems, virulence factors, meta-omics, rRNA, and related information. However, these databases only contain parts of available information and have high redundancy between them. In addition, bacterial big data integration technologies and advanced analysis tools are not rich enough. So, there is urgent need to integrate growing bacterial databases and develop more effective big data analysis tools for bacteria.

## Figures and Tables

**Table 1:** Database basic function comparison

	Online analyze tool	Help	Browse	Search	Download	
PATRIC	√(19)	√	√	√	√	{5}
EffectiveDB	√(1)	√	√	√	√	{5}
MPD	√(1)	√	√	√	√	{5}
VFDB	√(2)	√	√	√	√	{5}
PHI-base	√(1)	√	√	√	√	{5}
CARD	√(2)		√	√	√	{4}
MBGD		√	√	√	√	{4}
gcMeta		√	√	√	√	{4}
SILVA	√(4)	√	√	√	√	{5}
	7	8	9	9	9	

**Table 2:** Summary of bacteria databases

Database	Database description	Scope and scale	Microbiome data	Application/Tools available	Main data sources	Update frequency	Data construction methods
CARD	Bacterial antibiotic resistance-related gene, protein and mutation database	2678 reference sequences, involving 82 pathogens	No	Web interface, prevalence, resistomes & variants, BLAST, RGI, and RGI software	NCBI	Monthly	Text mining and database integration
EffectiveDB	Bacterial secreted proteins and secreted systems database	1699 bacterial genomes and their secreted proteins	No	Web interface, Prediction of secreted proteins and protein secretion systems, and EffectiveT3 software	NCBI, Simap, EggNOG	Quarterly	Generate by tools and database integration
MBGD	Microbial orthologous comparative genomic database	6318 genomes including 5861 bacteria, 254 archaea and 203 eukaryotes	No	Web interface, DomCluster, DomRefine, CGAT, and CoreAligner	NCBI, KEGG, PROSITE, DDBJ	Semiannual	Generate by tools and database integration
MPD	Bacterial genome and metagenomic database	41935 genomes of bacterial strains and 28950 metagenomic data from human and environmental samples	Yes	Web interface, and ANItools	NCBI, EMBL, Imicrobe, Meta-hit,MG-RAST, HMP, Ensembl	Yearly	Users upload and database integration
PATRIC	Comprehensive database of bacteria	236968 bacteria genomes, 3512 archaea genomes, 4719 virus genomes, and 10 eukaryotic hosts genomes	Yes	Web interface, genome assembly, genome annotation, comprehensive genome analysis, BLAST, similar genome finder, variation analysis, Tn-Seq analysis, phylogenetic tree building, genome alignment, metagenomic read mapping, taxonomic classification, metagenomic binning, expression data import, RNA-Seq analysis, protein family sorter, proteome comparison, comparative pathway, model reconstruction, and ID mapper	NCBI, KEGG, CARD, SEED, ResFinder, ArrarExpress, IntAct, BIND, DIP, MINT, BioGRID, PDB	Monthly	User upload and database integration
PHI-base	Pathogen and host interaction database	12467 pathogen-host interaction data with 266 pathogens and 199 hosts	No	Web interface, and PHI-BLAST	NCBI, Uploaded by users, EMBL, and web of science	Semiannual	Text mining, user upload, and databases integration
VFDB	Virulence factor database	32 genus of bacteria and 575 virulence factors in the core data set	No	Web interface, BLAST, and VFanalyzer	NCBI	Irregular updates	Text mining, generate by tools, and database integration
gcMeta	a Global Catalogue of Metagenomics platform	126602 samples, 153271 sequences, 146696 experiments and 77682 reports	Yes	Web interface, SRAtoolkit, ART, plRS, Bbtools, fastQC, cutadapt, Trimmomatic, fastp, dustmasker, DRISEE, Musket, SOAPec, LoRDEC, proovread, Quiver, FLASH, SOAPdenovo2, SPAdes, MetaVelvet, ALLPATH-LG, Meta-IDBA, MEGAHIT, RayMeta, CANU, CAP3, SSPACE, OPERA, QUAST, REAPR, CheckM, BUSCO, cufflinks, StringTie, Cuffdiff, Sailfish, Kallisto, DESeq2, Ballgown, Trinity, Oases, SOAPdenovo-Trans, PILER-CR, minced, tRNAscan SE, RNAmmer, Prodigal, Glimmer, GeneMark, FragGeneScan, XSTREAM, RepeatMasker, PRISM, LUMPY, Prokka, DFAST, InterProScan, PfamScan, QIIME(1,2), LEfSe, PICRUSt, MetaCV, k-SLAM, Kaiju, Centrifuge, DUDes, mOTU, StrainEst, Mash, sourmash, MetaPhlAn2, HUMAnN2, CONCOCT, MaxBin2, MetaBAT2, AbundanceBin, VirFinder, VirHostMatcher, orthoANI, CD-hit, MUMmer, BWA, Bowtie2, samtools, BLAST, BLAT, diamond, STAR, Tophat2, hisat2, blasr, R_plot_heatmap, R_plot_point, R_plot_PcoA, R_plot_barplot_fromtable	MG-RAST, EBI metagenomics and HMP and ongoing research projects such as CAS-CMI	Monthly	User upload, and databases integration
SILVA	High quality ribosomal RNA database	9470435 SSU Parc, 1312673 LSU Parc, 4945070 SSU Ref, 357845 LSU Ref, 659046 SSU Ref NR 99 and 123524 LSU Ref NR 99.	No	SILVAngs, SILVA Alignment, Classification and Tree (ACT) Service, SILVA Tree Viewer and ARB.	EMBL-EBI/ENA	Yearly	Generate by tools, and database integration
